# Bone sialoprotein facilitates anoikis resistance in lung cancer by inhibiting miR‐150‐5p expression

**DOI:** 10.1111/jcmm.70155

**Published:** 2024-10-28

**Authors:** Le Huynh Hoai Thuong, Chang‐Lun Huang, Yi‐Chin Fong, Chun‐Lin Liu, Jeng‐Hung Guo, Chih‐Ying Wu, Po‐I Liu, Chih‐Hsin Tang

**Affiliations:** ^1^ Graduate Institute of Biomedical Sciences China Medical University Taichung Taiwan; ^2^ Division of General Thoracic Surgery, Department of Surgery Changhua Christian Hospital Changhua Taiwan; ^3^ Department of Sports Medicine, College of Health Care China Medical University Taichung Taiwan; ^4^ Department of Orthopedic Surgery China Medical University Hospital Taichung Taiwan; ^5^ Department of Orthopedic Surgery China Medical University Beigang Hospital Yunlin Taiwan; ^6^ Department of Neurosurgery China Medical University Hospital Taichung Taiwan; ^7^ Department of Neurosurgery China Medical University Hsinchu Hospital Hsinchu Taiwan; ^8^ Department of Physical Therapy Asia University Taichung Taiwan; ^9^ Department of General Thoracic Surgery Asia University Hospital Taichung Taiwan; ^10^ Department of Pharmacology, School of Medicine China Medical University Taichung Taiwan; ^11^ Chinese Medicine Research Center China Medical University Taichung Taiwan; ^12^ Department of Medical Laboratory Science and Biotechnology, College of Medical and Health Science Asia University Taichung Taiwan

**Keywords:** anoikis resistance, BSP, lung cancer, miR‐150‐5p

## Abstract

Metastatic lung cancer is a highly prevalent cancer with a very low chance of long‐term survival. Metastasis at secondary sites requires that cancer cells develop anoikis resistance to survive during circulation. High levels of bone sialoprotein (BSP), a member of the small integrin‐binding ligand N‐linked glycoproteins (SIBLINGs), have been shown to promote the spread of lung cancer cells; however, the effects of BSP in anoikis resistance are largely unknown. In this study, we determined that BSP promotes anoikis resistance in lung cancer cells. BSP was also shown to promote the expression of E‐cadherin and vimentin (epithelial‐to‐mesenchymal transition markers, which have been utilized as indicators of anoikis resistance). It appears that BSP facilitates MMP‐14‐dependent anoikis resistance by inhibiting the synthesis of miR‐150‐5p and activating the ERK signalling pathway. Knockdown of BSP expression was shown to block lung cancer metastasis by lowering anoikis resistance *in vivo*. These results indicate that BSP is a promising target to deal with anoikis resistance and metastasis in human lung cancers.

## 1 INTRODUCTION

Lung cancer is among the most common forms of cancer worldwide^1^ and the third most common cancer in Taiwan.^2^ At the time of lung cancer diagnosis, metastasis is detected in roughly 50% of cases.^3^ Common metastasis sites include the nervous system, bone, liver, respiratory system and adrenal glands.^4^ Numerous therapies have been developed to inhibit lung cancer metastasis, including surgery, radiotherapy, targeted therapy, radiation therapy and chemotherapy^5^, ^6^; however, most of these methods can have harmful side effects.^7^ Efforts to develop less harmful treatment methods depend on a clear understanding of the mechanisms underlying lung cancer metastasis.

Anoikis is a form of programmed cell death (i.e., apoptosis) induced by the detachment of cells from the extracellular matrix (ECM) and is meant to remove cells that are not properly anchored. Metastasis at secondary sites requires that cancer cells escaping the tumour site develop anoikis resistance to survive passage through the lymphatic or circulatory systems.^8^, ^9^ Lung cancer has been linked to the development of anoikis resistance^10^ and has been shown to play a role in regulating the dissemination of non‐small cell lung cancer cells by modulating the composition of the ECM.^11^ A clear understanding of anoikis resistance in lung cancer cells is crucial to the development of methods aimed at impeding metastasis to distant organs.

Endogenous non‐coding RNAs (referred to as microRNAs; miRNAs) control physiological and pathological progression by binding to complementary sequences in their mRNA targets to prevent the expression of corresponding proteins.^12^ Researchers have determined that miRNAs regulate nearly a third of human genes,^13^ affecting a variety of biological processes, including cell development, differentiation and apoptosis.^14^ miRNAs have also been shown to control a variety of cellular functions in cancers, including drug resistance, invasion, metastasis and cell cycle progression.^15^, ^16^, ^17^

Bone sialoprotein (BSP) is a member of the small integrin‐binding ligand N‐linked glycoprotein (SIBLING) family. High BSP expression levels have been shown to promote the metastasis of lung cancer cells.^18^ In prostate and breast cancer cell lines, elevated BSP expression levels promote cell survival, migration and invasion.^19^ High BSP levels in the serum of lung cancer patients are correlated with the likelihood of metastasis^20^ and distant metastasis.^21^ One study reported that BSP was associated with the dissemination of lung cancer into bone.^22^ In a previous study, we determined that BSP induces the expression of metalloproteinase‐14 (MMP‐14) in lung cancer cells *in vitro* and *in vivo*, thereby increasing the likelihood of osteolytic bone metastasis.^23^ Additionally, MMP‐14 has been shown to play a role in anoikis resistance in colorectal cancer.^24^ Nonetheless, the role of BSP in anoikis resistance during lung cancer metastasis remains a mystery. In the current study, we determined that BSP enhances anoikis resistance in human lung cancer cells by up‐regulating MMP‐14 production. The ERK signalling pathway and miR‐150‐5p synthesis mediate the BSP‐induced promotion anoikis resistance. Knocking down BSP expression was shown to diminish lung cancer metastasis and anoikis resistance *in vivo*. Thus, BSP and miR‐150‐5p are promising targets to deal with anoikis resistance and metastasis in human lung cancer.

## 2 MATERIALS AND METHODS

### 2.1 Materials

BSP recombinant protein was acquired from R&D systems (Minneapolis, MN, USA). siRNAs against MMP‐14 and control siRNAs were purchased from Dharmacon (Lafayette, CO, USA) (The sequences are listed in Table S1). BSP shRNA was purchased from the National RNAi Core Facility (RNAi Core, Academia Sinica, Taiwan) (The sequences are listed in Table S2). All other chemicals were acquired from Sigma‐Aldrich (St. Louis, MO, USA).

### 2.2 Cell culture

Human lung cancer cell lines A549 and CL1‐5 were purchased from the American Type Culture Collection (Manassas, VA, USA). A549 cells were cultured with RPMI medium (Gibco, Waltham, MA, USA) supplemented with 10% FBS.^25^ CL1‐5 cells were grown in a DMEM medium (Gibco, Waltham, MA, USA) supplemented with 10% FBS. After cell growth achieved 80% confluence, the cells were maintained in a humidified incubator at 37°C under 5% CO_2_.

### 2.3 Anoikis assay

To inhibit cell attachment, lung cancer cells (5 × 10^4^/well) were seeded on 24‐well plates precoated with poly‐HEMA (P3932, Sigma‐Aldrich, St. Louis, MO, USA) for 24 h.^26^ The cells were then treated with BSP (0–30 ng/mL) for various durations (1, 2, or 3 days). Aggregated cells were collected, trypsinized daily and counted using a 0.4% Trypan blue solution (T8154, Sigma‐Aldrich, St. Louis, MO, USA). The anoikis‐resistant cell count was defined as the number of live cells observed under a microscope. The percentage of anoikis‐resistant cells was calculated in accordance with previously established methods.^27^

$$\frac{\text{number of living cells}}{5\times 10^{4}\;\left(\text{number of seeding cells}\right)}\times 100$$

### 2.4 Western blot analysis

Total cell lysates were obtained using RIPA buffer. The Pierce™ BCA Protein Assay Kit was used to determine protein concentrations in each cell lysate (#23225; Thermo Scientific, Waltham, MA, USA) using the electrophoresis and transfer procedure protocols from previous investigations.^28^, ^29^ Briefly, membranes were blocked using 5% nonfat milk for 1 h prior to probing using the following antibodies: p‐ERK, ERK, vimentin, anti‐mouse secondary antibodies and anti‐rabbit secondary antibodies (Santa Cruz Biotechnology, Inc., CA, USA); and MMP‐14 (MyBioSource, San Diego, CA, USA), E‐cadherin (Abcam, Cambridge, MA, USA), and β‐actin antibodies (Sigma‐Aldrich, St. Louis, MO, USA). Luminescence was detected using the iBright™ Imaging System (#CL1500, Thermo Scientific, Waltham, MA, USA).^30^

### 2.5 Real‐time quantitative PCR

Total RNA was isolated from A549 and CL1‐5 cells and then reverse‐transcribed to complementary DNA (100 ng) using an M‐MLV RT kit (Invitrogen, Thermo Fisher Scientific, USA). qPCR was performed in accordance with a predefined methodology (qPCR primers sequences are listed in Tables S3 and S4).^31^, ^32^

### 2.6 Database analysis

From The Cancer Genome Atlas (TCGA) database (GSE45142), we compiled a comprehensive dataset containing gene expression information specific to lung cancer patients, with a focus on evaluating E‐cadherin and vimentin expression levels. Subsequently, we acquired and subjected this dataset to in‐depth analysis, investigating gene expression dynamics across different stages of lung cancer, particularly distinguishing between metastatic and non‐metastatic cases based on the dataset annotations.

To further investigate pathways involved in tumour progression, we analysed the GSE45142 dataset. Using Ingenuity Pathway Analysis (IPA), we explored the complex networks of pathways linked to tumour development. For a more detailed gene analysis, we also used the GEO2R tool from the Gene Expression Omnibus (GEO) database to identify genes with statistically significant changes (*p* < 0.05, fold change between −2 and 2). These genes were then input into the IPA software to reveal key pathways involved in tumour progression.

### 2.7 Transfection

The control and miR‐150‐5p mimic or inhibitor (Allbio Science Incorporate, Taiwan) was transfected into A549 and CL1‐5 cells using Lipofectamine 2000 (Invitrogen) in accordance with the manufacturer's instructions.

### 2.8 Luciferase activity

Luciferase plasmids of MMP14 3′‐UTR plasmids containing wild‐type (WT) or mutant (MUT) miR‐150‐5p binding sites were obtained from MDBio, Inc. (Taipei, Taiwan). The luciferase plasmids were transfected into lung cancer cells using Lipofectamine 2000, after which the cells were treated with BSP for an additional 24 h. Luciferase activity was monitored using a dual luciferase assay system (Promega) in accordance with the manufacturer's instructions.^33^

### 2.9 Animal model

A549 and A549 BSP shRNAs (1 × 10^6^) were injected into the caudal artery in the tail of six‐week‐old male nude mice purchased from BioLASCO Taiwan Co., Ltd. (Taipei, Taiwan).^34^ After 8 weeks, the IVIS® Spectrum imaging system was used to determine the *in vivo* mass of the tumour. After the mice were humanely sacrificed, tumour tissues were subjected to immunohistochemistry (IHC) and haematoxylin and eosin staining. The animal study had been approved by the Institutional Animal Care and Use Committee of China Medical University.

### 2.10 IHC staining

Tumour cell sections were deparaffinized using xylene and rehydrated using ethanol. IHC staining was performed using a NovoLink Polymer System (Leica Microsystems) in accordance with the manufacturer's instructions. Human BSP, E‐cadherin, vimentin, or MMP‐14 antibodies were added at a dilution of 1:200 and then incubated at 4°C overnight. Sections were counterstained using haematoxylin. In scoring the IHC results using Image J software, we accounted for the percentage of positive detections as well as the intensity of staining.^35^, ^36^

### 2.11 Statistics

All values are expressed as the mean ± standard deviation. All differences between experimental and control groups were assessed for significance using the Student's *t*‐test. All data analysis and charting of results were performed using GraphPad Prism 8.0 software. Between‐group differences were considered significant if the *p*‐value was <0.05.

## 3 RESULTS

### 3.1 BSP promotes resistance to anoikis in lung cancer cells

**FIGURE 1:**
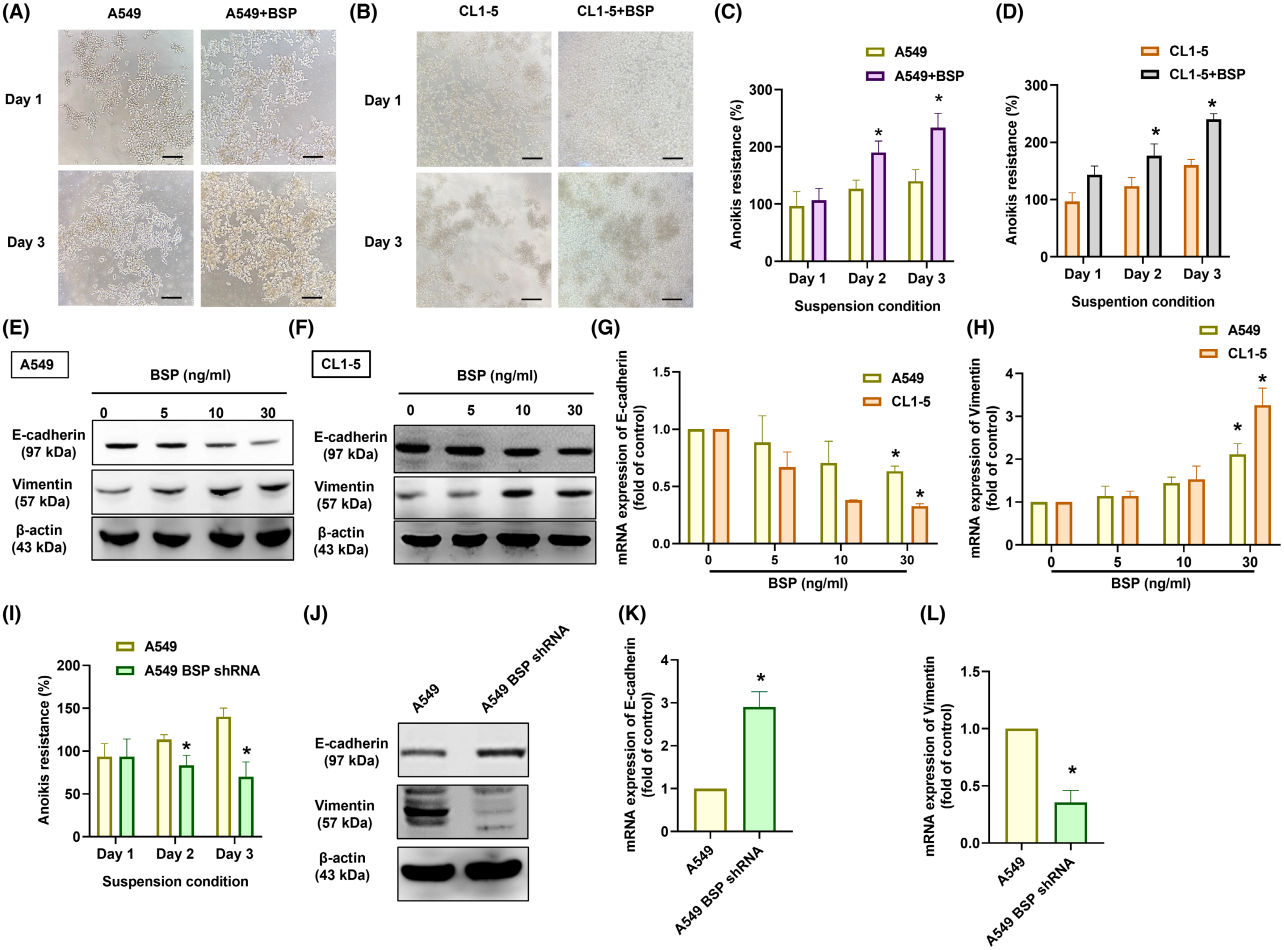
BSP promoted anoikis resistance in lung cancer cells: (A–D) Anoikis resistance after incubating cells with BSP for indicted time intervals (scale bar 200 μm); (E–H) Western blot analysis and qPCR results showing E‐cadherin and vimentin expression levels after treating cells with BSP (I–L) Anoikis resistance and E‐cadherin and vimentin expression in indicated lung cancer cells. *p < 0.05 compared with the control; #*p* < 0.05 compared with the BSP‐treated group.

**FIGURE 2:**
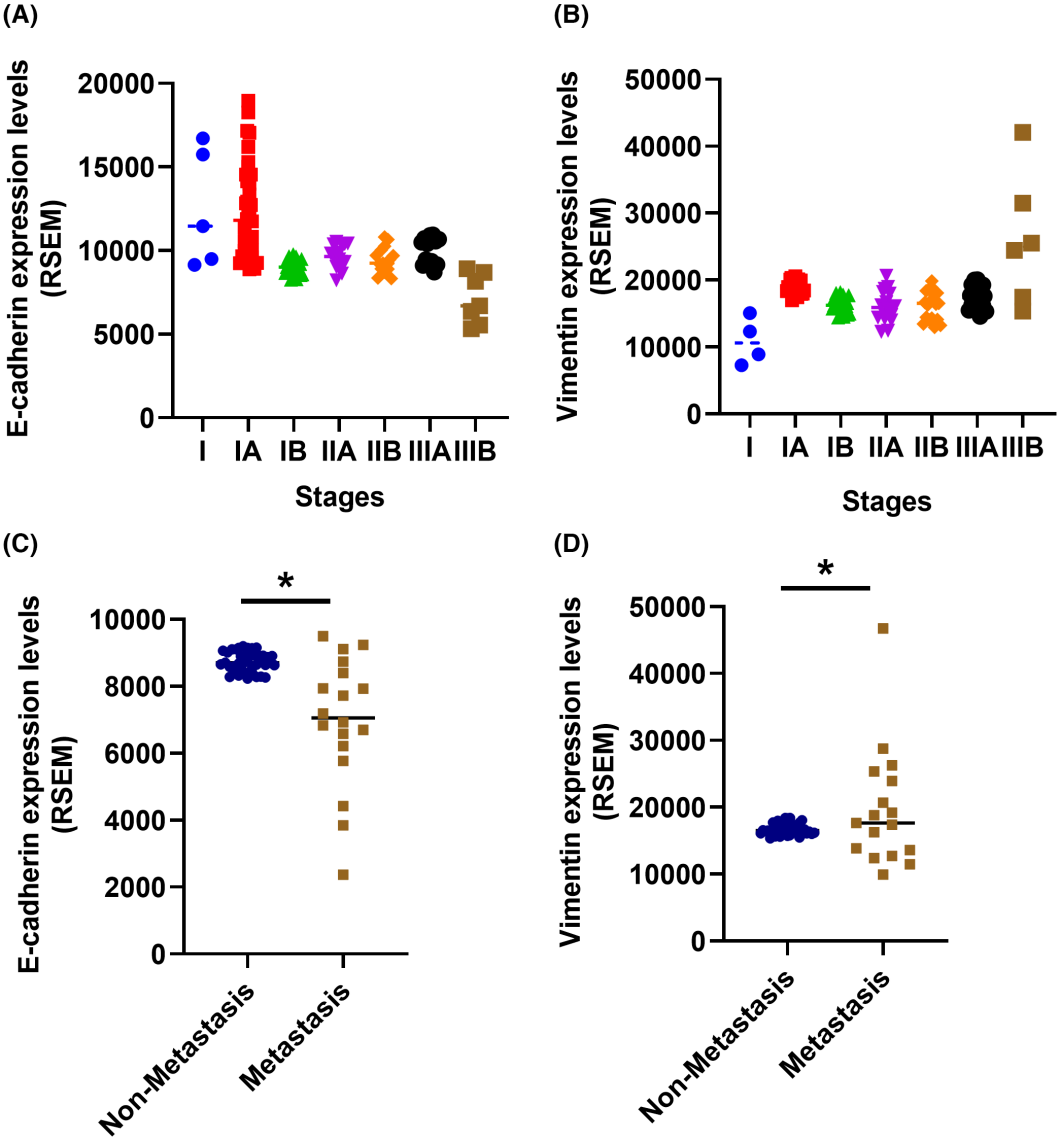
E‐cadherin and vimentin expression in human lung cancer patients: E‐cadherin and vimentin mRNA levels in lung cancer tissues from The Cancer Genome Atlas. *p < 0.05 compared with the non‐metastasis patients.

High BSP levels have been shown to promote metastasis in lung cancer patients,^21^, ^22^ and researchers have reported that anoikis resistance is associated with lung cancer metastasis.^10^ In a previous study, BSP stimulation enhanced invasion ability in two human lung cancer cell lines.^23^ We therefore sought to determine whether BSP mediates resistance to anoikis in lung cancer. On a low‐adherence culture plate, PolyHEMA was used to culture A549 and CL1‐5 cells with anoikis resistance,^27^, ^37^ as indicated by survival for 3 days in adherence‐ and anchorage‐independent conditions through the formation of micro‐tissues (Figure 1A,B). BSP stimulation was shown to enhance anoikis resistance in both cell lines (Figure 1A–D), whereas BSP knockdown using shRNA reduced anoikis resistance (Figure 1I). Anoikis‐resistant cells are those that hijack the EMT process to attain anoikis resistance after losing cell–cell attachment or ECM.^38^ Thus, it is possible to use EMT markers (e.g. E‐cadherin or vimentin) as markers of anoikis resistance. Furthermore, there were significant correlations observed between BSP and both E‐cadherin and Vimentin (Figure S1). Treating lung cancer cells with BSP inhibited the protein and mRNA expression of E‐cadherin and promoted vimentin expression (Figure 1E–H), whereas inhibiting BSP expression had the opposite effects (Figure 1J–L). Our analysis of the TCGA data revealed significantly lower E‐cadherin mRNA levels in high‐stage lung cancer tissues, along with significantly higher vimentin mRNA levels (Figure 2A,B). In addition, similar results have found in metastasis patients than non‐metastasis patients (Figure 2C,D). Taken together, it appears that BSP promotes anoikis resistance in human lung cancer cells.

### 3.2 BSP enhances MMP‐14‐dependent anoikis resistance via the ERK pathway

**FIGURE 3:**
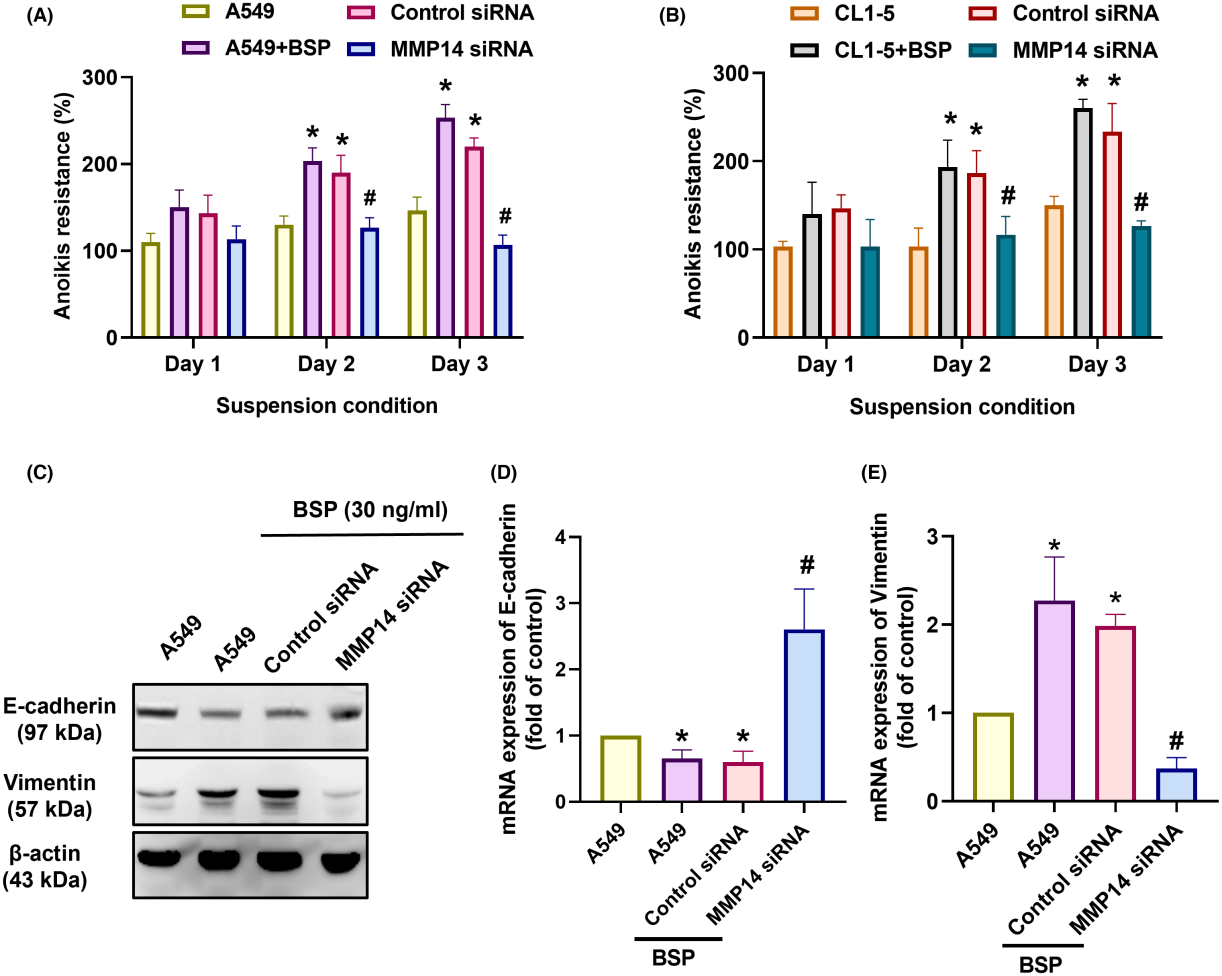
BSP enhanced MMP‐14‐dependent anoikis resistance in lung cancer cells: (A, B) Anoikis resistance in cells transfected with MMP‐14 siRNA; (C–E) Western blot analysis and qPCR results showing E‐cadherin and vimentin expression in cells transfected with MMP‐14 siRNA. *p < 0.05 compared with the control; #*p* < 0.05 compared with the BSP‐treated group.

**FIGURE 4:**
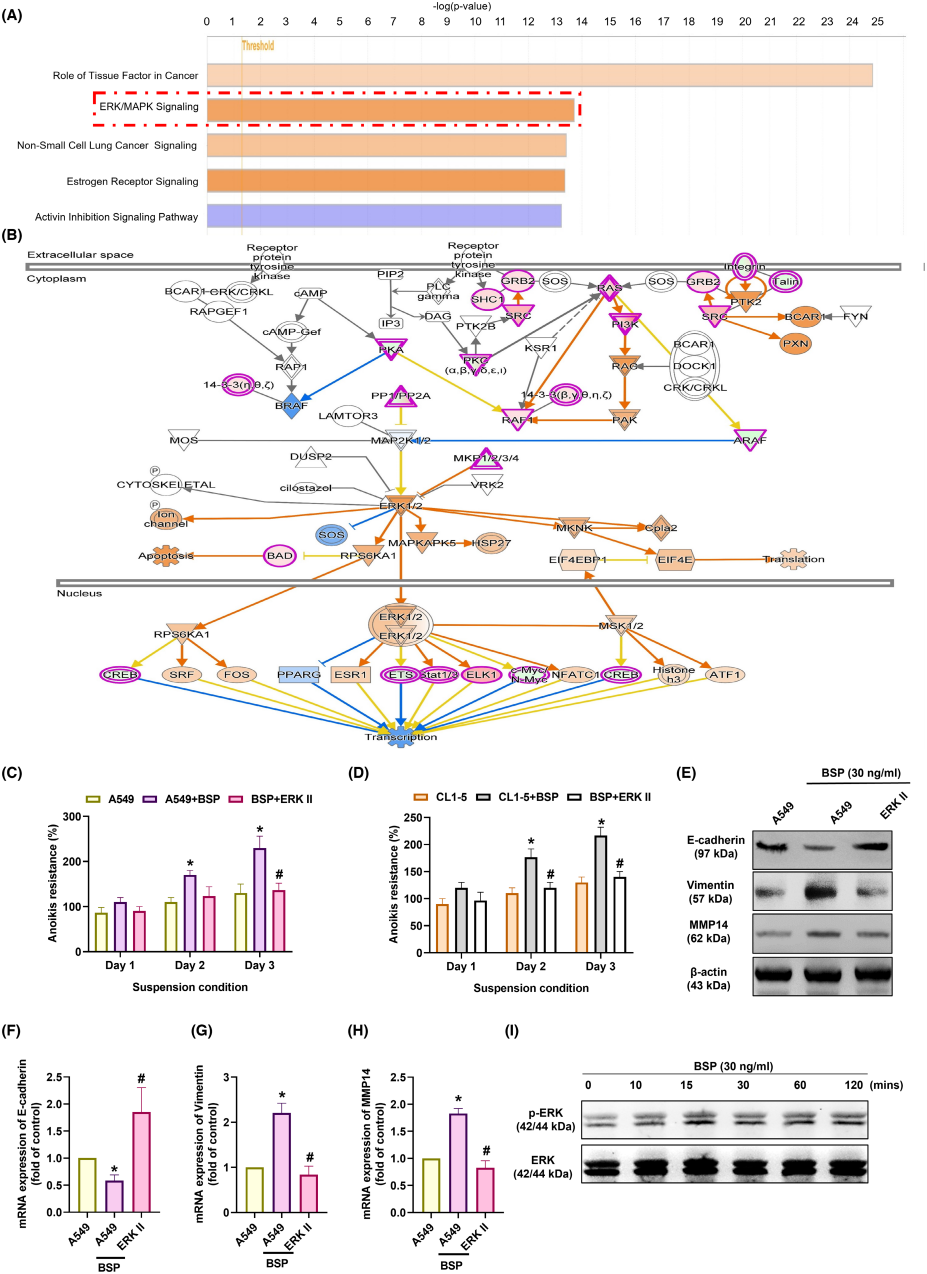
BSP promoted MMP‐14‐dependent anoikis resistance via the ERK pathway: (A, B) Ingenuity Pathway Analysis pathway enrichment figure showing pathways in the GSE45142 database that were significantly upregulated; (C, D) Anoikis resistance ability in cells treated with ERK inhibitor (ERK II); (E–H) Western blot analysis and qPCR results showing E‐cadherin, vimentin, and MMP‐14 expression levels in cells treated with ERK II; (I) Western blot analysis showing ERK expression in cells treated with BSP for indicated time intervals. *p < 0.05 compared with the control; #*p* < 0.05 compared with the BSP‐treated group.

It has been reported that MMP‐14 plays a regulatory role in the development of osteolytic bone metastasis in cases of lung cancer.^23^ We therefore sought to determine whether MMP‐14 plays a role in BSP‐induced anoikis resistance. Transfection with MMP‐14 siRNA was shown to antagonize BSP‐facilitated anoikis resistance (Figure 3A,B). MMP‐14 siRNA also rescued BSP‐controlled changes in EMT markers (Figure 3C–E). Thus, it appears that BSP promotes MMP‐14‐dependent anoikis resistance in lung cancer. We next sought to elucidate the mechanism underlying anoikis resistance by analysing signalling pathways in the GSE45142 database using IPA software. Our analysis revealed a significant correlation between the ERK/MAPK signalling pathway, which is the top signalling in lung cancer (Figure 4A,B). It has been reported that BSP activates ERK signalling in prostate and breast cancer cells.^19^ Incubating cells with an ERK inhibitor (ERK II) reduced BSP‐enhanced anoikis resistance and changes in EMT markers (Figure 4C–H), while stimulating cells with BSP activated ERK phosphorylation (Figure 4I). Taken together, it appears that the ERK signalling pathway regulates BSP‐induced anoikis resistance in human lung cancer.

### 3.3 miR‐150‐5p regulates BSP‐induced MMP‐14‐dependent resistance to anoikis

**FIGURE 5:**
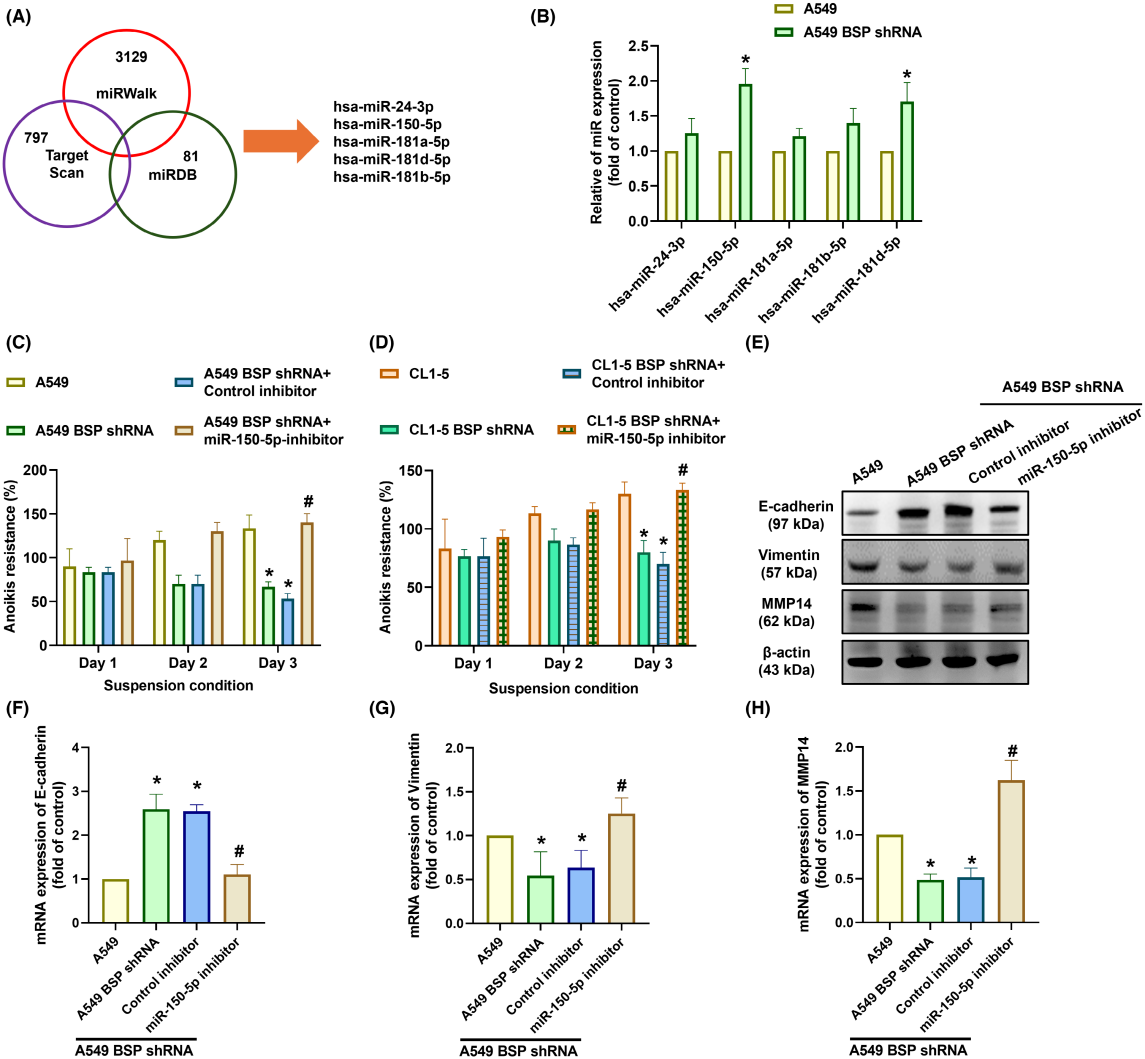
miR‐150‐5p regulated BSP‐facilitated anoikis resistance: (A) Predicted miRNA interference with MMP‐14 transcription derived by combining three datasets; (B) qPCR results showing miRNA expression in indicated cells; (C, D) Anoikis resistance in cells transfected with miR‐150‐5p inhibitor; (E–H) Results of Western blot analysis and qPCR showing E‐cadherin, vimentin, and MMP‐14 expression levels in cells transfected with miR‐150‐5p inhibitor. *p < 0.05 compared with the control; #*p* < 0.05 compared with the BSP‐treated group.

**FIGURE 6:**
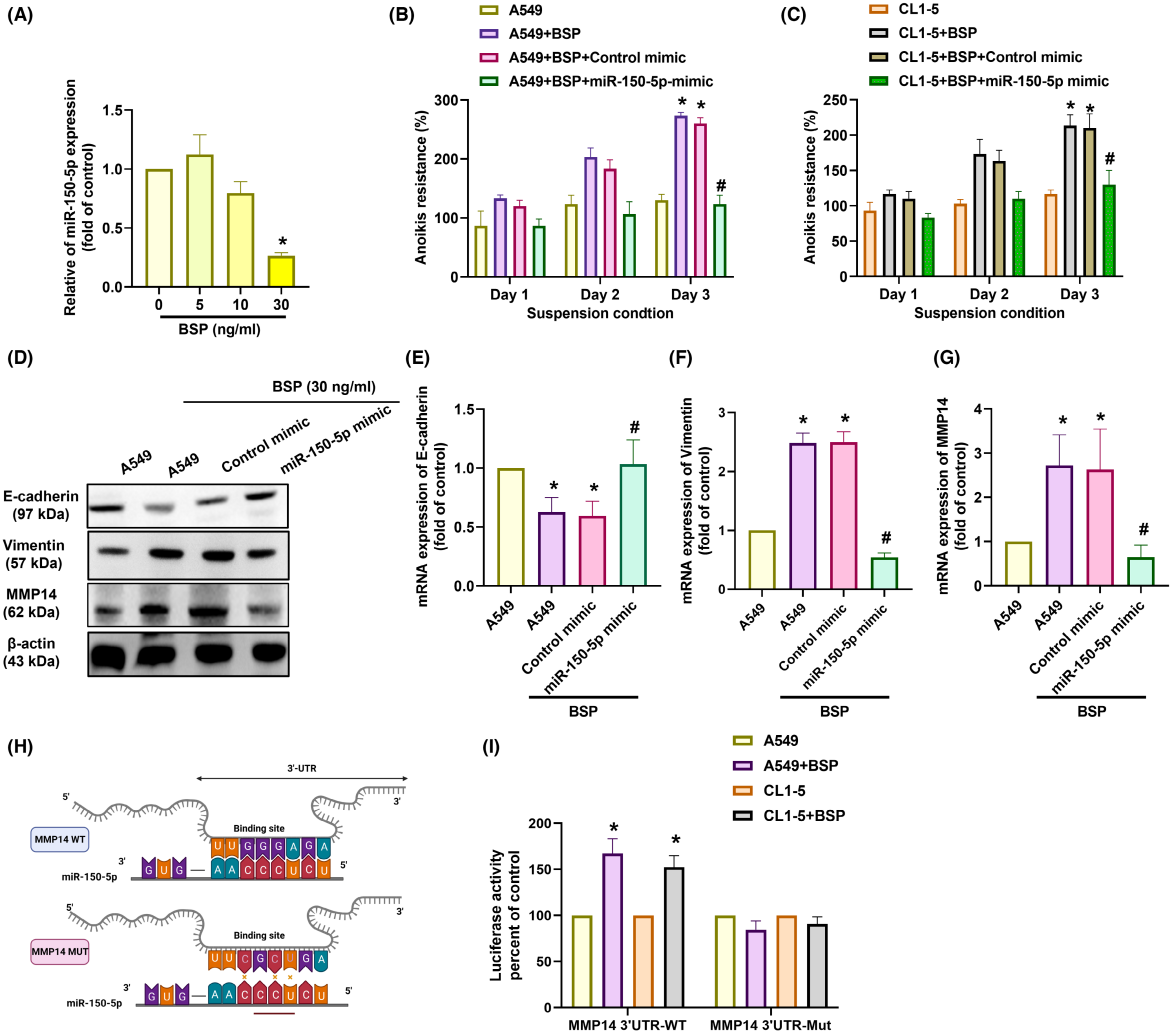
BSP promoted anoikis resistance by inhibiting miR‐150‐5p expression: (A) qPCR results showing miR‐150‐5p expression levels in cells treated with BSP; (B, C) Anoikis resistance in cells transfected with miR‐150‐5p mimic; (D–G) Results of Western blot analysis and qPCR showing E‐cadherin, vimentin, and MMP‐14 expression levels in cells transfected with miR‐150‐5p mimic; (H) Schematic 3'UTR representation of human MMP‐14 containing the miR‐150‐5p binding site; (I) Relative luciferase activity in cells transfected with the indicated luciferase plasmid prior to treatment with BSP *p < 0.05 compared with the control; #*p* < 0.05 compared with the BSP‐treated group.

Researchers have reported that miRNAs regulate anoikis in non‐small cell lung cancer.^39^ In the current study, our investigation of three miRNA databases (miRWalk, TargetScan and miRDB) revealed five miRNAs that may regulate MMP14 expression (Figure 5A). miR‐150‐5p expression was significantly higher in A549 BSP‐shRNA cells than in A549 cells (Figure 5B). We subsequently used a miRNA inhibitor to elucidate the role of miR‐150‐5p in the observed effects of transfection with a miR‐150‐5p inhibitor reduced anoikis resistance, changes in EMT markers, and MMP‐14 expression in A549 BSP‐shRNA cells (Figure 5C–H). By contrast, BSP stimulation of A549 cells reduced miR‐150‐5p expression in a concentration‐dependent manner (Figure 6A). Transfection with a miR‐150‐5p mimic also blocked BSP‐regulated anoikis resistance, changes in EMT markers, and MMP‐14 expression in A549 and CL1‐5 cells (Figure 6B–G). The direct effect of miR‐150‐5p on MMP‐14 transcription was assessed by generating luciferase reporter vectors containing WT or MUT 3'‐UTR of MMP‐14 mRNA (Figure 6H). BSP was shown to induce WT activity but not MUT MMP‐14 3'‐UTR luciferase activity (Figure 6I). These data indicate that BSP enhanced anoikis resistance by increasing MMP‐14 synthesis via miR‐150‐5p inhibition.

### 3.4 Inhibiting BSP suppressed anoikis resistance and lung cancer metastasis *in viv* *o*

**FIGURE 7:**
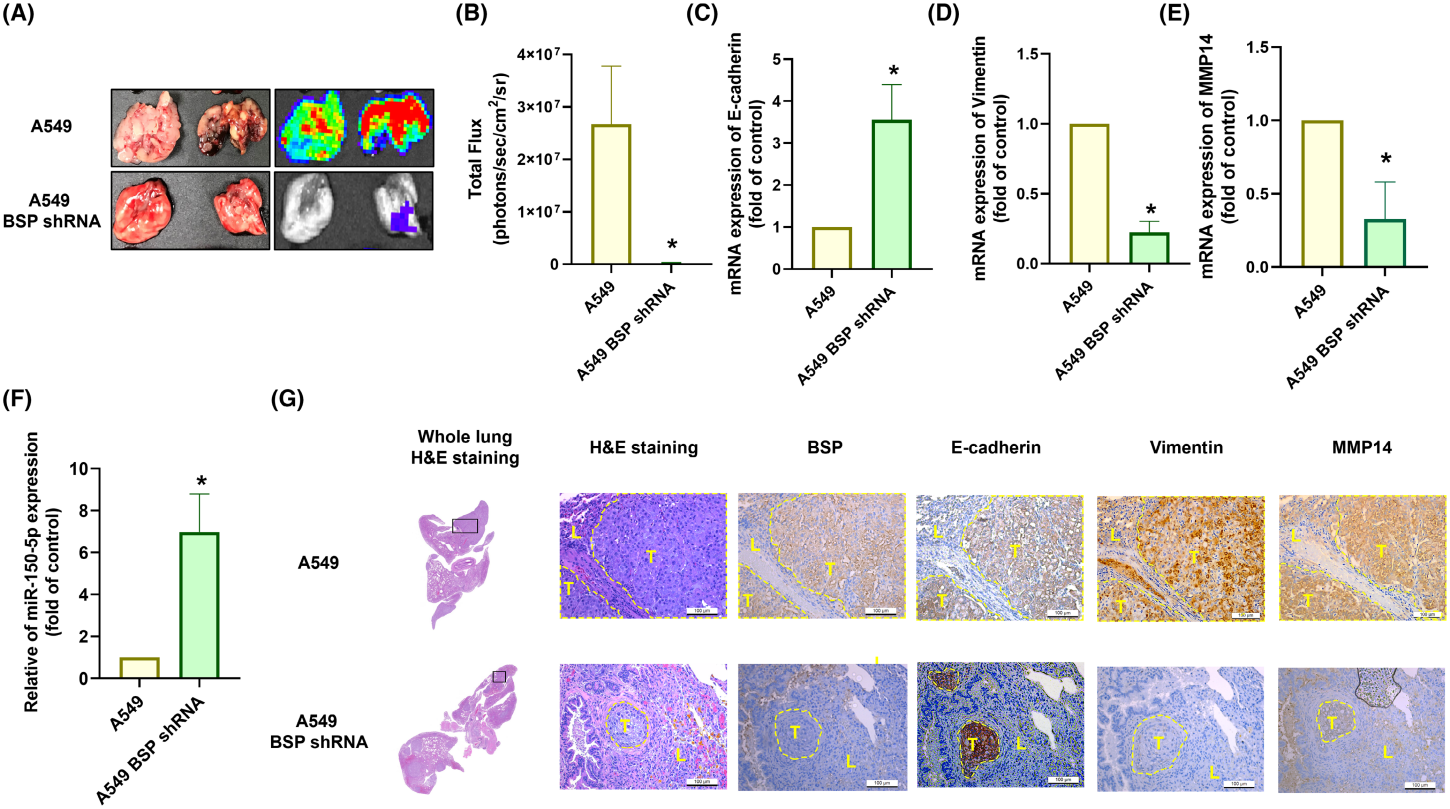
BSP knockdown inhibited lung cancer metastasis and anoikis resistance *in vivo*. (A, B) Representative bioluminescent images used to quantify lung metastasis at 8 weeks after the injection of indicated cells into mice; (C–F) qPCR results showing E‐cadherin, vimentin, MMP14, and miR‐150‐5p expression levels in blood; (G) Haematoxylin & eosin staining and IHC staining showing BSP, E‐cadherin, vimentin, and MMP‐14 levels in lung tissue. *p < 0.05 compared with the control.

We investigated the therapeutic effects of BSP on anoikis resistance and metastasis in lung cancer by establishing an animal model, which involved injecting A549 cells or A549 BSP‐shRNA cells through the caudal artery of nude mice. At 8 weeks post‐injection, bioluminescence signals in metastatic sites of the lung were markedly lower in the A549 BSP‐shRNA group than in the A549 group (Figure 7A,B). Furthermore, the serum mRNA expression levels of E‐cadherin and miR‐150‐5p were higher in the BSP‐knockdown group, while vimentin and MMP‐14 were lower (Figure 7C–F). In A549 BSP‐shRNA cells, haematoxylin and eosin analysis of whole lung revealed the inhibition of control cells in forming metastatic tumour nodules. In the A549 BSP‐shRNA group, IHC staining also revealed lower levels of BSP, vimentin and MMP‐14 protein expression and the higher E‐cadherin protein expression (Figure 7G). These results indicate that inhibiting BSP expression reduced lung cancer metastasis and anoikis resistance *in vivo*.

## 4 DISCUSSION

Lung cancer is a leading cause of cancer death worldwide.^40^ Many people who undergo surgery for early‐stage lung cancer experience recurrence within 5 years after surgery, and most newly diagnosed individuals present local progression or metastasis.^41^ Hypoxia, angiogenesis and cancer cell migration are just a few of the issues that contribute to lung cancer metastasis.^42^, ^43^, ^44^ We previously reported that BSP facilitates the development of osteolytic bone metastasis in cases of human lung cancer.^23^ In the current study, we determined that anoikis resistance also contributes to BSP‐mediated lung cancer metastasis. It appears that BSP induces MMP‐14‐dependent anoikis resistance by inhibiting the synthesis of miR‐150‐5p and activating the ERK signalling pathway.

When epithelial and endothelial cells lose attachment to the ECM or nearby cells, they undergo a specific type of apoptosis, referred to as anoikis, a physiological mechanism involved in tissue repair and organ development.^45^ Resistance to anoikis in cancer cells is typically associated with increased aggressiveness, including enhanced invasive and migratory capabilities. Anoikis resistance is believed to be a prerequisite for metastasis, involving the passage of tumour cells through the circulatory system to promote the growth of secondary tumours in distant organs.^8^ In the current study, our use of the PolyHEMA model to mimic anoikis resistance *in vitro* revealed that BSP stimulation promoted resistance to anoikis in two lung cancer cell lines. Likewise, BSP inhibition was shown to suppress anoikis resistance. Our *in vivo* model also demonstrated that BSP knockdown decreased lung cancer metastasis, presumably by reducing resistance to anoikis. BSP represents a promising pharmacological target for the development of small molecules or antibodies aimed at treating lung cancer metastasis.

During the EMT process, cells undergo a number of genetic and morphological changes in order to shed their epithelial properties and develop a more undifferentiated mesenchymal phenotype. EMT is widely known for granting stem‐like traits and cell plasticity, which contribute to the acquisition of invasive phenotypes by reducing cell–cell adhesion and promoting metastatic capacity.^46^, ^47^ EMT also permits the evasion of anoikis, a particular form of apoptotic cell death brought on by a lack of cell‐matrix connections.^48^ One trait of stem‐like cells is resistance to anoikis, which is thought to be a key factor in the spread of cancer cells and their ability to metastasize.^49^ In this study, two EMT markers were used as anoikis resistance markers: E‐cadherin and vimentin. BSP inhibition was shown to promote protein and mRNA expression of E‐cadherin and inhibit vimentin expression *in vitro* and *in vivo*. Our Cancer Genome Atlas results revealed that E‐cadherin levels were markedly lower in lung cancer patients than in healthy controls, while vimentin levels were markedly higher. In addition, the E‐cadherin and vimentin expression are associated with metastasis of lung cancer patients. Our results confirm that the EMT process is involved in BSP‐mediated anoikis resistance and metastasis in lung cancer.

ERK is a signalling pathway critical to the modulation of cell motility and metastasis in human cancer.^19^ For instance, WNT5A promotes MMP‐14 expression and cell motility in human osteosarcoma cells via the ERK pathway.^50^ CTHRC1 promotes the progression of oesophageal squamous cell carcinoma by activating ERK signalling.^51^ Our results obtained from the GSE dataset using IPA software confirmed that the ERK signalling pathway is associated with the top signalling pathway (ERK/MAPK signalling). Indeed, ERK pharmacological inhibitor were shown to diminish BSP‐induced anoikis resistance. BSP stimulation was also shown to enhance ERK activation, which is required for the BSP‐mediated synthesis of MMP‐14 and anoikis resistance.

Abnormal regulation of miRNA expression in lung cancer has been shown to promote disease progression.^12^ miRNAs have been used as biomarkers in tumour‐associated diagnoses and treatment.^14^ They have also been shown to regulate the ability of human cancer cells to evade anoikis.^52^ However, there is little evidence to support assertions that the MMP‐14‐miRNA axis plays a regulatory role in anoikis resistance in lung cancer. In the current study, the miR‐150‐5p mimic reduced the BSP‐mediated synthesis of MMP‐14 and anoikis resistance, while BSP‐knockdown had the opposite effects *in vitro* and *in vivo*. Researchers have reported that miR‐150‐5p hinders the motility of hepatoma cell motility via MMP‐14 regulation.^53^ Suetsugu et al. reported that a miR‐150‐5p mimic reduces MMP‐14 production and the aggressiveness of squamous cell carcinoma.^54^ Taken together, it appears that the MMP‐14/miR‐150‐5p axis plays a key role in regulating tumour metastasis.

**FIGURE 8:**
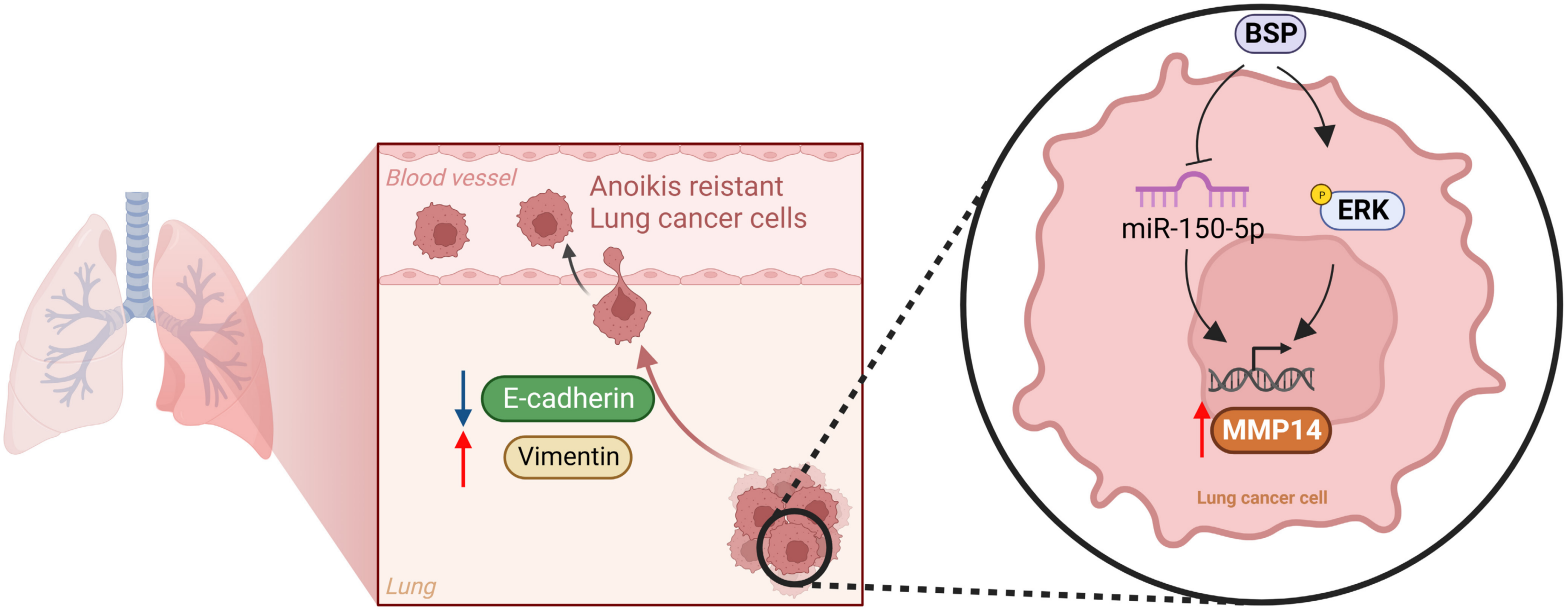
Schematic illustration showing signalling pathways involved in BSP‐facilitated anoikis resistance in lung cancer. BSP facilitated MMP‐14‐dependent anoikis resistance in human lung cancer by inhibiting the synthesis of miR‐150‐5p and activating the ERK signalling pathway.

To summarize, the current study demonstrated that BSP promotes MMP‐14‐dependent resistance to anoikis in human lung cancer cells by inhibiting the synthesis of miR‐150‐5p and activating the ERK signalling pathway (Figure 8). These results demonstrate that BSP and miR‐150‐5p are strong candidates for the development of novel anti‐metastasis agents.

## AUTHOR CONTRIBUTIONS

**Le Huynh Hoai Thuong:** Conceptualization (equal); data curation (equal); formal analysis (equal); writing – original draft (equal). **Chang‐Lun Huang:** Conceptualization (equal); data curation (equal). **Yi‐Chin Fong:** Methodology (equal); software (equal). **Chun‐Lin Liu:** Methodology (equal); software (equal). **Jeng‐Hung Guo:** Investigation (equal); methodology (equal); resources (equal). **Chih‐Ying Wu:** Software (equal); visualization (equal). **Po‐I Liu:** Software (equal); supervision (equal); visualization (equal). **Chih‐Hsin Tang:** Conceptualization (equal); project administration (equal); supervision (equal); writing – review and editing (equal).

## CONFLICT OF INTEREST STATEMENT

The authors have no financial or personal relationships that could inappropriately influence this research.

## Supporting information


Data S1.

## Data Availability

The data that support the findings of this study are available on request from the corresponding author upon reasonable request.
