# Supplementary material for: Bone sialoprotein facilitates anoikis resistance in lung cancer by inhibiting miR‐150‐5p expression
Source: J Cell Mol Med. 2024 Oct 28;28(20):e70155. doi: 10.1111/jcmm.70155 (PMC11514928; doi:10.1111/jcmm.70155)
Supplement: Supplementary file 1 — Data S1. [file JCMM-28-e70155-s001.docx]

**Supplementary Tables**

**Supplementary Table S1. siRNA used in this study.**

| **Gene** | **Species** | **Source** | **Catalog No.** |
| --- | --- | --- | --- |
| **MMP14** | Human | Dharmacon (2650 Crescent Dr, Lafayette, USA) | L-004145-00-0005 |
| **Control** | Human | Dharmacon (2650 Crescent Dr, Lafayette, USA) | D-001810-10-05 |

**Supplementary Table S2. shRNA used in this study.**

| **Name** | **Sequence** |
| --- | --- |
| BSP shRNA | CCGGGAGACTTCAAATGAAGGAGAACTCGAGTTCTCCTTCATTTGAAGCTCTTTTTTG |

**Supplementary Table S3. Primers used in this study.**

| **Gene** | **Forward** | **Reverse** |
| --- | --- | --- |
| MMP14 | GGCTACAGCAATATGGCTACC | GATGGCCGCTGAGAGTGAC |
| Vimentin | GCCCTAGACGAACTGGGTC | GGCTGCAACTGCCTAATGAG |
| E-cadherin | GGTGCTCTTCCAGGAACCTC | TAAGCGATGGCGCATTGTA |
| GAPDH | AATGGACAACTGGTCGTGGA | CCCTCCAGGGATCTGTTTG |

**Supplementary Table S4. miRNA primers used in this study.**

| **Micro RNA** | **Sequence** |
| --- | --- |
| miR-24-3p | TGCCTACTGAGCTGATATCAGT |
| miR-150-5p | TCTCCCAACCCTTGTACCAGTG |
| miR-181a-5p | AACAT TCAAC GCTGT CGGTG AGT |
| miR-181b-5p | AACAT TCATT GCTGT CGGTG GGT |
| miR-181d-5p | AACAT TCATT GTTGT CGGTG GGT |

**Supplementary Figure**


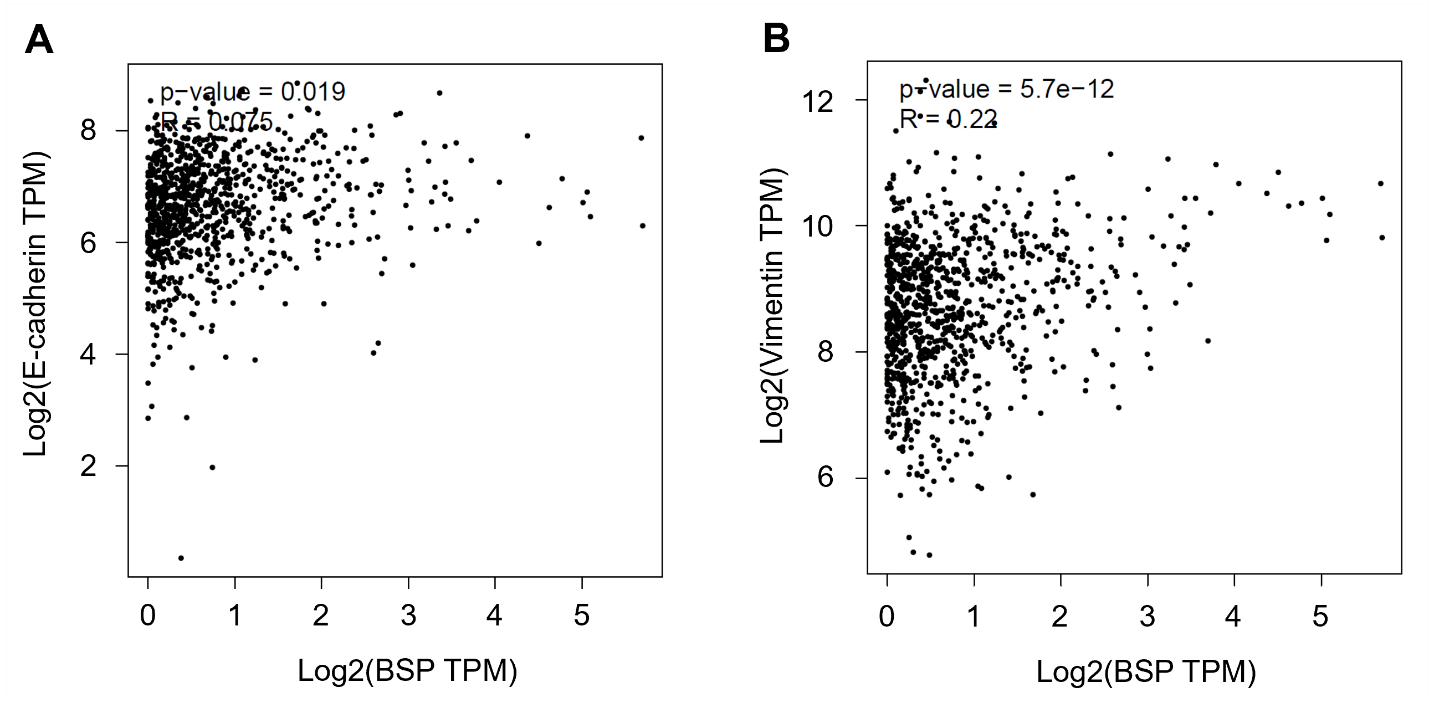


**Supplementary Figure S1. Correlation between BSP and E-cadherin or Vimentin.** Data from the GEPIA2 database show the correlation between BSP and E-cadherin (A) and BSP and Vimentin (B) in lung adenocarcinoma and lung squamous cell carcinoma.
